# Chalcone constituents pulincisones A-F isolated from *Pulicaria incisa* with NQO1 inducer activity

**DOI:** 10.1038/s41598-025-15999-2

**Published:** 2025-08-20

**Authors:** Ahmed R. Hamed, Tarik A. Mohamed, Sally A. Abdel-Halim, Mohamed H. Abd El-Razek, Sayed A. El-toumy, Maureen Higgins, Krystyna Skalicka-Woźniak, Albena T. Dinkova-Kostova, Paul W. Paré, Mahmoud M. Sakr, Mohamed-Elamir F. Hegazy

**Affiliations:** 1 Chemistry of Medicinal Plants Department, National Research Centre, 33 El-Bohouth St., Dokki, Giza, 12622 Egypt; 2 Natural Compounds Chemistry Department, National Research Centre, 33 El-Bohouth St., Dokki, Giza, 12622 Egypt; 3 Department of Chemistry of Tannins, National Research Centre, 33 El-Bohouth St., Dokki, Giza, 12622 Egypt; 4 Division of Cellular and Systems Medicine, School of Medicine, Jacqui Wood Cancer Centre, University of Dundee, Dundee, DD1 9SY Scotland, UK; 5 Department of Chemistry of Natural Products, Medical University of Lublin, Lublin, Poland; 6 Department of Chemistry & Biochemistry, Texas Tech University, Lubbock, TX 79409 USA; 7 Department of Plant Biotechnology, Institute of Genetic Engineering and Biotechnology, National Research Centre, 33 El-Bohouth St., Dokki, Giza, 12622 Egypt

**Keywords:** *Pulicaria incisa* (Lam.) DC., Asteraceae, Dihydrochalcone, Chemopreventive, Antioxidant, NQO1, Natural products, Metabolic pathways

## Abstract

Previously unknown chalcones such as *β*’-chalcanone-*α*,*β*-diols and a *β*-hydroxydihydrochalcone, named pulincisone A-F as well as known chalcone analogs 3,4,2’,4’,6’,7,8-heptahydroxy-7(8)-dihydrochalcone (**7**) and 4,2’,6’-trihydroxy-4’-methoxydihydrochalcone (**8**) were isolated from *Pulicaria incisa* (Lam.) DC. and assayed for cancer preventative activity. Structures were identified by spectroscopic methods, including HRESIMS, 1D and 2D NMR experiments. Pulincisone A and B, are epimers, at stereocenter C-8. A structural revision resulted in pulicisone A (**1**) and D (**4**) replacing pulichalconoids B and C, that were previously reported from the same species. Isolated chalcones were assessed for chemopreventive potential as inducers of NAD(P)H: quinone oxidoreductase 1 (NQO1). Compounds **2**, **3**, **5**, and **6** induced NQO1, using a Prochaska assay. NQO1 protein expression was detected by Western blotting analysis. Compound **6** among four active compounds had the highest potency as NQO1 inducers. Molecular docking analysis of the NQO1 Keap1 Kelch domain suggests Nrf2-dependent induction. These data indicate that pulincisone F (**6**) is a promising bioactive antioxidant (NQO1-inducer) with superior docking scores with the Keap1-Nrf2 complex compared to the reference NQO1 inducer sulforaphane (− 8.26 versus − 5.08 kcal mol^-^, respectively).

**Supplementary Information:**

The online version contains supplementary material available at 10.1038/s41598-025-15999-2.

## Introduction

*Pulicaria incisa* (Lam.) DC. is a rare desert plant that belongs to the Asteraceae family^1^; it is multipurpose medicinal shrub native to Egypt^2^. It is used by Egyptian Bedouins to treat heart diseases and an infusion of the plant has been evaluated for use as a functional beverage to inhibit and treat brain injuries and neurodegenerative disease^2^. It has been utilized in folk medicine as an anti-inflammatory, antioxidant, and antimicrobial agent^3^. Phytochemical analysis of *P. incisa* (Lam.) DC. indicates the presence of alkaloids, flavonoids, chalcones (pulichalconoid B and pulichalconoid C)^4^, tannins, ellagic acids, saponins, sterols, terpenes, cardiac glycosides, essential oils and fatty acids^5,6^.

Chalcones are flavonoids that are distinguished by an absence of a C ring in the skeleton, resulting in an open-chain classification^7^. The two aromatic rings are connected by an *α*,*β*-unsaturated ketone system Chalcones possess promising biological activity, distinctive physical properties and are the precursor to all flavonoids^8^. Structure-activity relationships have been established by adding specific substituents to one or both aryl rings^9^. This uncommon family of flavonoid metabolites are known as either *α*- and *β*-(dihydrochalcone) chalcones^10^. Chalcone constituents have been shown to attenuate cancer progression via activation of a Nrf2/ARE pathway which in turn upregulates NQO1 expression^11^.

Nrf2 is a transcription factor that is negatively regulated by Kelch-like ECH-associated protein 1 (KEAP1). Under basal conditions, Nrf2 is bound to KEAP1 which serves as a substrate adaptor protein for a Cullin-3/Rbx1 ubiquitin ligase and mediates the continuous ubiquitination and proteasomal degradation of Nrf2^12^. With cancer progression, KEAP1 is inactivated which in turn induces Nrf2^13^. Keap1 interacts with Nrf2 through the C-terminal Kelch domain. Altered KEAP1 gene regulation leads to NRF2 protein accumulation and induces robust transactivation of cytoprotective genes encoding for detoxification and antioxidative enzymes such as NAD(P)H: quinone oxidoreductase 1 (NQO1) and heme oxygenase-1 (Ho-1, encoded by the Hmox1 gene)^14^. Many chemo-preventive agents target the Keap1/Nrf2 protein complex and inactivate Keap1, causing Nrf2 stabilization and induction of downstream target genes encoding cytoprotective proteins. The Kelch domain represents a high-quality model for the superfamily of eukaryotic Kelch repeat proteins^15^. X-ray crystallography has resolved the Kelch structure in humans with a resolution of 1.85 Å^15^.

The present study reports on the identification of newly characterized chalcone derivatives from *P. incis* (Lam.) DC. Since electrophilic chalcones with an *α*,* β*-unsaturated ketone moiety have been shown to bind to Keap1 thiol groups and prevent conjugation of Keap1 with Nrf2, the isolated chalcones were assayed for Nrf2 induction by monitoring the downstream product NQO1^16^. To probe chalcone mode of action, *in* *silico* docking studies were performed focusing on the Kelch domain.

## Results and discussion

### Chemical characterization

**Fig. 1:**
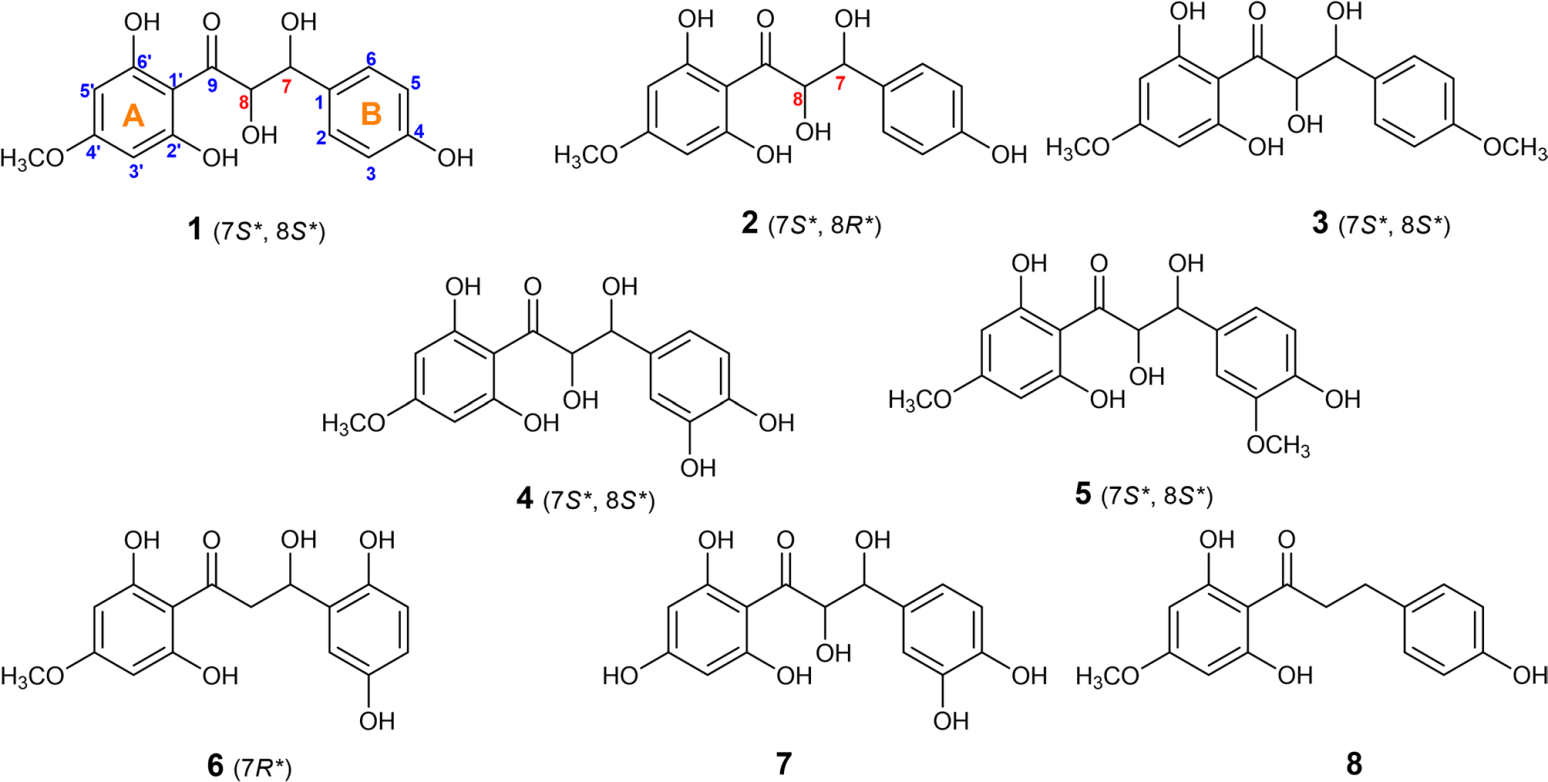
Structure of compounds **1**-**8**.

The plant extract purified by column chromatography resulted in six new metabolites *βˋ*-chalcanone-*α*,*β*-diols (**1**–**5**), and *β*-hydroxydihydrochalcone (**6**) as well as two previously identified compounds: 3,4,2’,4’,6’,7,8-heptahydroxy-7(8)-dihydrochalcone **(7)**^17^ and 4,2’,6’-trihydroxy-4’-methoxydihydrochalcone **(8)**^18,19^ (Fig. 1). Chemical structures were elucidated based on spectroscopic methods, including HRESIMS, 1D and 2D NMR experiments (Fig. S1-S48).

### Identification and structural elucidation

**Fig. 2:**
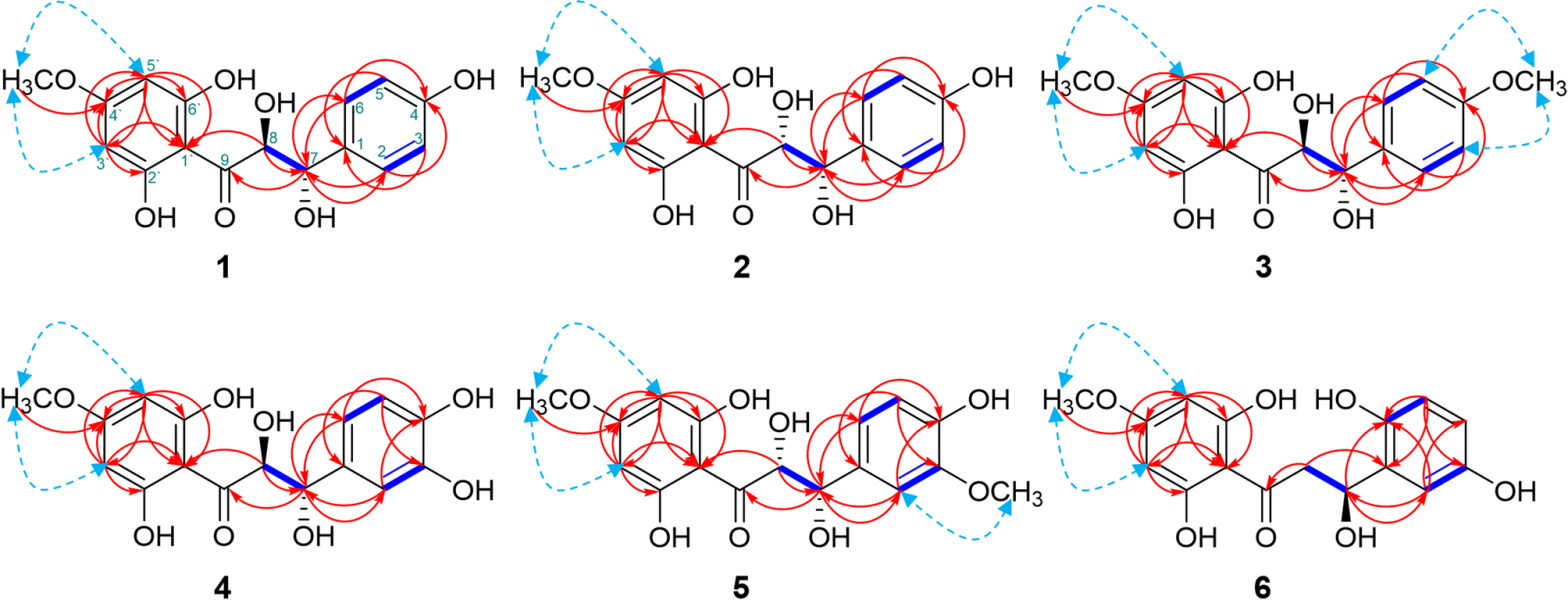
Observed ^1^H-^1^H COSY (blue colour straight line), HMBC (red colour curved arrow) and NOESY (sky blue colour curved arrow) correlations of compounds **1-6**.

Pulincisone A (**1**) was purified as yellowish oil, with $\left[ a \right]_{D}^{{25}}$= + 9.6 (*c* = 0.01, MeOH). The molecular formula C_16_H_16_O_7_ was identified by HRESIMS (Fig. S8) with an ion peak at *m/z* 301.0702 [M-H-H-18]^−^, calcd. for C_16_H_13_O_6_^−^, 301.0712), suggesting nine degrees of unsaturation in accordance with the ^13^C NMR spectral data (Table 1; Figure S2).The IR spectrum showed absorptions due to hydroxyl groups (3577, 3496 and 3271 cm^− 1^), a carbonyl group (1705 cm^− 1^) and a pair of phenyl bands (2947 and 1451 cm^− 1^)^20^. Analysis of the^1^H, ^13^C NMR, DEPT and HMQC spectra (Table 1; Figure S1-S3 & S6) revealed the presence of six aromatic protons: [four of which were typical for an AA’BB’ system, indicating a *para*-substitution in the aryl ring B (*δ*_H_ 7.33 (2H, d, *J* = 8.2 Hz, H-2,-6)/ *δ*_C_ 129.1; *δ*_H_ 6.81 (2H, d, *J* = 8.2 Hz, H-3,- 5)/ *δ*_C_ 114.8), and the other two protons exhibited an AX pattern system showing a *meta*-coupling in the aryl ring A (*δ*_H_ 5.99 (1H, d, *J* = 2.7 Hz, H-3’)/ *δ*_C_ 93.7; *δ*_H_ 6.04 (1H, d, *J* = 2.7 Hz, H-5’)/ *δ*_C_ 94.7], an AB spin coupling system was assigned to a pair of carbolic protons [(*δ*_H_ 4.95 (1H, d, *J* = 12.0 Hz, H-7)/ *δ*_C_83.7; *δ*_H_ 4.53 (1H, d, *J* = 12.0 Hz, H-8)/ *δ*_C_72.4], a methoxy [*δ*_H_ 3.77 (3H, s, 4’-OMe)/ *δ*_C_ 55.0], in addition to six quaternary *sp*^2^ carbons [*δ*_C_ 101.3 (C-1’); 127.8 (C-1); 157.9 (C-4); 163.0 (C-2’); 163.7 (C-6’); 168.5 (C-4’); and finally a benzoyl carbonyl 197.7 (C-9)]. The ^13^C NMR (Table 1) and DEPT spectra displayed a total of 16 carbon signals that were attributed to a C_6_-C_3_-C_6_ system plus a methoxy group. A dihydrochalcone skeleton was proposed based on NMR and UV maxima at 333, 312, 287, 227, and 203 nm^21^. ^1^H-^1^H COSY (Figure S4) showed four spin systems H-3’/H-5’; H-2/H-3; H-5/H-6; and H-7/H-8. From the mutual coupling in the ^1^H-^1^H COSY, one-2,4,6 trioxygenated substitution characteristic of an A ring *para*-disubstituted aryl B ring along with vicinal existence for both oxymethines H-7 and H-8. Based on the ^13^C NMR data along with nine degrees of unsaturation, a planner structure was proposed with bicyclic rings. An AB spin system ^1^H, ^13^C NMR) was visible in place of the common chalcone *trans* coupling AB spin system characteristics for an *α*,*β*-unsaturation-*γ*-keto; this confirmed the enolization of the chalcone-*β*,*β’*-dione^22,23^. The ^13^C NMR diagnostic feature was a shielded resonance signal at *δ*_C_ 101.3, assigned to C-1’ due to the absence of an olefinic core in the main chalconoid skeleton. Key HMBC correlations (Fig. 2 & S5) included: H-7/ C-2, C-6, C-9; H-8/ C-1, C-1’, C-9; H-2, H-6 /C-4, C-7; H-3, H-5/C-1; H-3’, H-5’/C-1’. Both of aromatic methines at *δ*_C_ 93.7 (C-3’) and 94.7 (C-5’) were assigned to *met*a positions within the tetra-substituted oxyaryl A ring. C-5’ absorbed more in the deshielded position than C-3’ resonance, possibly due to carbon orientation differences or chelating of the neighboring phenolic hydroxyl group. Based on ^3^*J*_CH_^2^, *J*_CH_ of H-3’ and H-5’correlations in the HMBC spectrum with (C-2’), (C-4’), (C-6’) and (C-1’) in combination with ^2^*J*_CH_ *δ*_H_ 3.77/*δ*_C_55.0, besides the supporting of the NOESY cross peak (Figure S7) of *δ*_H_ 3.77 (4’-OMe) to *δ*_H_ 5.99 (H-3’) and *δ*_H_ 6.04 (H-5’) allowed for assigning the methoxy group at C-4’ (A ring) (Fig. 2)^18^. The oxyaryl quaternary carbons of the A ring, were assigned to a methoxylated C-4’ (*δ*_C_ 168.5) and were shifted downfield due to an electron withdrawal effect by the *para* carbonyl group (C-9). The other moiety of the chalcone framework belonged to the *para*-disubstituted aryl ring B. This was indicated by HMBC cross peak between H-3, H-5 to C-4/C-1 and H-2, H-6 to C-4. To the best of our knowledge, chemical shifts of the A ring are not affected by substitution of B ring, and vice versa. In addition, to the assignment of the carbonyl resonance, it appears at the most downfield chemical shift at *δ*_C_ 197.7. The presence of the 6’-OH group moves the signal downfield by 3–5 ppm relative to the corresponding 6’-OMe, owing to intermolecular hydrogen bond interactions^24^. Finally, the focus was on the bridge carbons relative to the propane-2,3-diol-1-one junction. The propane-2,3-diol-1-one junction was deduced from its downfield shift in the ^13^C NMR spectrum and HMBC correlations from H-7/ C-2, C-6, C-9 and H-8/ C-1, C-1’, C-9. In fact, the ^1^ H and ^13^C NMR spectra were very similar to those of **7**, previously known as Cilicione-A, isolated from *Thymus cilicicus* (Labiatae). A minor difference in the substitution pattern of the A ring suggested **1** was hydroxylated at C-4‘^17^. The singlet at *δ*_H_ 3.77/*δ*_C_ 55.0, associated with aromatic methoxy of the A ring (4’-OMe) in **1**, disappears with Cilicione-A, which is consistent with this hypothesis. Therefore, the 1,3-diarylpropane skeleton was modified by the presence of a 1,3-diarylpropane-2,3-diol-1-one.

**Table 1:** ^1^H NMR (600 MHz) and ^13^C NMR (125 MHz) spectral data of pulicisone A-C (**1**-**3**) in CD_3_OD (*δ* in ppm, *J* values in parentheses).^a^

No	Pulincisone A (1)		Pulincisone B (2)		Pulincisone C (3)	
	δ_H_(J in Hz)	δ_C_	δ_H_(J in Hz)	δ_C_	δ_H_(J in Hz)	δ_C_
1		127.8		126.7		127.9
2	7.33 d (8.2)	129.1	7.33 d (8.2)	128.5	7.34 d (8.2)	129.2
3	6.81 d (8.2)	114.8	6.77 d (8.2)	114.6	6.94 d (8.2)	109.7
4		157.9		157.3		146.8
5	6.81 d (8.2)	114.8	6.77 d (8.2)	114.6	6.94 d (8.2)	109.7
6	7.33 d (8.2)	129.1	7.33 d (8.2)	128.5	7.34 d (8.2)	129.2
7	4.95 d (12.0)	83.7	5.36 d (2.7)	81.7	4.98 d (12.0)	83.6
8	4.53 d (12.0)	72.4	4.18 d (2.7)	71.7	4.57 d (12.0)	72.5
9		197.7		195.7		196.0
1҆		101.3		101.2		100.9
2҆		163.0		163.0		163.0
3҆	5.99 d (2.7)	93.7	6.04 d (2.7)	93.7	6.01 d (2.7)	94.8
4҆		168.5		168.4		168.9
5҆	6.04 d (2.7)	94.7	6.08 d (2.7)	94.6	6.06 d (2.7)	95.6
6҆		163.7		164.4		163.7
4’-OMe	3.77 s	55.0	3.80 s	55.0	3.78 s	56.0
4-OMe					3.86	56.0

^a^Assignments were based on ^1^H-^1^H-COSY, HMQC and HMBC experiments.

**Scheme 1:**
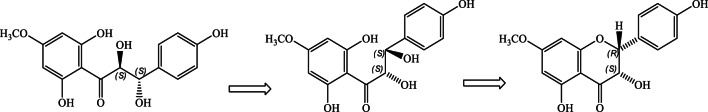
Change in the configuration occurs during the *S*_*N*_^2^-Type of Mitsunobu reaction.

**Fig. 3:**
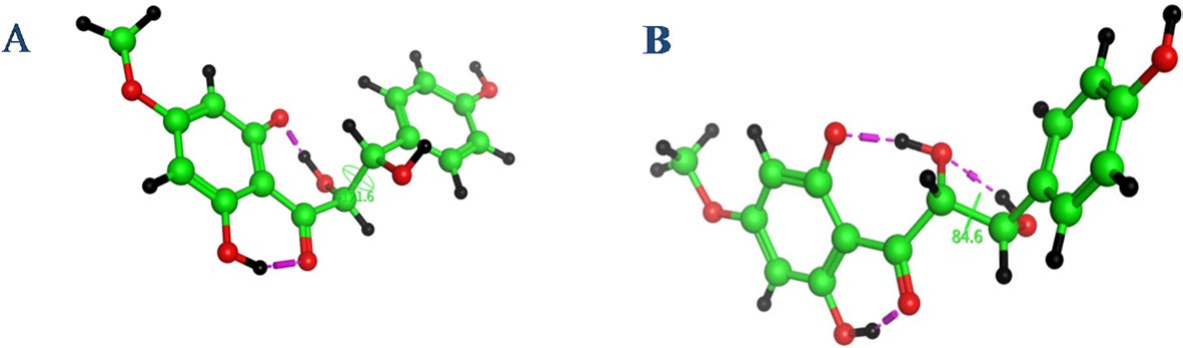
**(A)** With two intermolecular hydrogen bonds (ArHO….OH &ArHO…CO) and a dihedral angle of almost 171.6º between H-7 and H-8, the three-dimensional configuration skeleton of **1** demonstrated their anti-orientation, establishing the *thero*-7,8 conformer. (**B**) The three-dimensional configuration skeleton of **2** showed their anti-orientation, producing the *erthero*-7,8 conformer, with three intermolecular hydrogen bonds (ArHO…OH, ArHO…CO & HO…HO) and a dihedral angle of almost 84.6º between H-7 and H-8.

The ^1^H and ^13^C NMR spectral data along with the same sign of the optical rotation of **1** were very similar to those of rhusopolyphenol E, which was previously reported from the bark of *Rhus verniciflua*^25^ with details elucidation of its absolute configuration indicating that, **1** shares the same carbon framework main skeleton. A minor difference between them was in the substitution of both aromatic rings A and B. For determining absolute configuration, **1** had a significant resistance to crystallization and is significantly laborious to synthesize for Mosher’s method. Proton coupling in the *anti*-oriented (*J* = 8–9 Hz), compared to *syn*-isomers (*J* = 2–3 Hz), has been established in closely related a cyclic polyol derivatives^25,26^ which allowed us to demonstrate the relative configuration of **1** in the 7,8-*thero* form. In a report described by Mitsunobu, cyclization^27,28^ standard *SN*^2^-type Mitsunobu reaction conditions were employed to obtain the enantio selective flavonol (Scheme 1), that was confirmed to have the absolute configuration of (2*S*,3*S*). A classical observation that intramolecular hydrogen bonding (Fig. 3A)^20^ and the coupling constant of (*J* = 12.0 Hz, see Table 1) between H-7 and H-8 were found in the ^1^H NMR spectra (Table 1) were used to deduce *thero*-configurations at the two stereocenters (C-7 and C-8) in the aliphatic chain junction. The characteristic signal for H-7 was identified at *δ*_H_ 4.56 (1 H, d, *J* = 12.0 Hz)^17,25^ and using this as a point of reference, *δ*_H_ 4.95 (d, *J* = 12.0 Hz) was identified as H-8 by COSY (Figure S4). The dihedral angle measured at the two stereocenters in the aliphatic chain, which came out to be 171.6º (Fig. 3A), was again found to be in accord with the large coupling constant (*J* = 12.0 Hz) and suggested that the *thero*-configuration was involved. The relative stereochemistry assignment was identified based in part on the coupling constant as well as the previously reported NMR chemical shift for similar metabolites^17,25^. The large coupling constant between H-7 and H-8 (12.0 Hz) indicated the *α*-configuration of H-7 and the *β*-configuration of H-8^17,25^. Thus, the structure of **1** was assigned as (2S*, 3S*)-1-(2,6-dihydroxy-4-methoxyphenyl)-2,3-dihydroxy-3-(4-hydroxyphenyl) propan-1-one and giving a trivial name of pulincisone A (Fig. 1).

Pulincisone B (**2**) was isolated as a yellowish oil. Based on HRESIMS (Figure S16) ^13^C, and DEPT NMR spectra the molecular formula was determined to be C_16_H_16_O_7_. The MS, UV, IR, and NMR spectral data (Figure S9-S15) were like **1**, even in several chromatographic systems. They had comparable 1D and 2D NMR spectra, with similar resonances observed. Table 1 summarizes the chemical shift and coupling differences are shown (Table 1). Compounds **2** and **1** were identified as diastereo isomers as they had the same covalent structures but different physical characteristics. Due to the intermolecular hydrogen bond (HO—OH) (Fig. 3B)^20^ between the two vicinal hydroxyl groups, which is most likely the result of little differences in intermolecular hydrogen bonding. It appears that contributes significantly to the bending modes of the (1,2-diol) structure configuration in the aliphatic bridge. Furthermore, instead of being dextrorotatory for **1**:$~\left[ a \right]_{D}^{{25}}$= + 9.6 (MeOH; *c* = 0.01), the optical rotation of **2** changed to levorotatory: $\left[ a \right]_{D}^{{25}}$ = − 17.7 (*c* = 0.05, MeOH)^29–31^. Considering the spectral data, it is confirmed that both **1** and **2** have the same chalconoid skeleton. The existence of nearby chiral centers at C-7 and C-8 is what causes the stereoisomerism in these molecules (**1** and **2**). Synthetic structures were identified by NMR, MS, and chemical modifications; in this work it was identified that the compounds were diastereo isomers^31–34^. Our results and the preceding study’s conclusions are comparable in this regard. Nonetheless, they diverge in how the two diastereo isomers are assigned for the *erythro*-and *threo*-forms^31,35,36^. The detected ^13^C NMR chemical shift differences between **1** and **2**, together with different^1^H NMR coupling constants between H-7 and H-8, suggested an opposite configuration of the C-8 stereogenic center^37^. Further support for the suggested structure arises from the comparison of ^13^C NMR chemical shifts of the C-7,8-*thero* conformation, that shifted downfield, whereas the *erytho*-isomer is shifted upfield^38,39^. A previous study showed that *erythro*- and *threo-*forms of this type of molecule may be distinguished from one another using the magnitude of the coupling constant between H-7 and H-8^40^. Empirically, the *erythro*-form is represented by a small coupling constant with H-7, H-8 values having a lower limit of around 3.2 Hz, while the *threo*-form is represented by larger coupling constant for H-7, H-8 with values of 8–12 Hz^33^. The trend for a larger coupling constant in *anti*-orientation protons (*J* = 12.0 Hz) of **1** compared to their *syn*-isomer **2** (*J* = 2.7 Hz) has been well established and closely related to acyclic propane-2,3-diol-1-one, which allowed us to suggest that the conformation of **2** should be *erytho* of the vicinal diols comparison with **1**, which is the *thero* isomer. The dihedral angle between H-7 and H-8 that varies from 60 to 180ᴼ, can be connected with this range of potential *J* values^41^. The dihedral angle measured between them yields a value of 84.6ᴼ, which is appropriate for the 2.7 Hz coupling constant (Fig. 3B). According to these data, **1** and **2** are diastereomers, epimeric at C-8 and having the same configuration at C-7. The structure of **2** is therefore re-assigned as (2R*,3S*)-1-(2,6-dihydroxy-4-methoxyphenyl)-2,3-dihydroxy-3-(4-hydroxyphenyl) propan-1-one, with a trivial name of pulincisone B (Fig. 1).

Pulincisone C (**3**) was isolated as a yellowish oil with an optical rotation of $\left[ a \right]_{D}^{{25}}$ = + 19.1 (*c* = 0.02, MeOH). The molecular weight measured by HRESIMS (Figure S24) was *m/z* 301.0723 [M-H-MeOH]^−^, and calcd. for C_17_H_18_O_7_, 334.1053, with nine degrees of unsaturation. When compared to **1**, the existence of a methoxy group connected to one of the two aryl rings was proposed. The spectra were similar to **1** with a distinct substitution pattern for the aryl ring B, in addition to the anticipated signals of the methoxy group at *δ*_H_ 3.86 and *δ*_C_ 56.0 (Figure S17-S19). Indeed, COSY, HSQC and HMBC spectra (Fig. 3B & S20-S22), contained signals diagnostic of an AA’BB’ system, with a *para*-substitution in the aryl ring B and a methoxy group connected to C-4. In the NOESY spectrum (Fig. 2 & S23), both H-3 and H-5 showed significant correlations to 4-OMe. The influence of the methoxy group at C-4 (B ring) shifted H-3 and H-5 downfield by Δ*δ*_H_ = + 0.13 ppm. Furthermore, the position of the methoxy group on C-4 shifted upfield for C-3, C-4, and C-5. 4-OMe shows two different interactions involving the increasing electron release on C-4, one shifted C-4 upfield (Δ*δ*_C_= -11.1 ppm) by a *β*-effect and the other on C-3,C-5 by a γ- or *ortho*-effect shifted to upfield (Δ*δ*_C_= -5.1 ppm), setting a clear comparison with the^13^C NMR spectrum of the *p*-hydroxylated form of the ring B of **1**. Consequently, the structure of **3** was assigned as (2S*,3S*)-1-(2,6-dihydroxy-4-methoxyphenyl)-2,3-dihydroxy-3-(4-methoxyphenyl) propan-1-one with a trivial name of pulincisone C (Fig. 1).

Pulincisone D (**4**) was isolated as a brownish oil with an optical rotation of$~\left[ a \right]_{D}^{{25}}$ = + 12.8 (*c* = 0.05, MeOH). The chemical formula was deduced to be C_16_H_16_O_8_ with nine degrees of unsaturation based on HRESIMS (Figure S32) with an ion peak at *m/z* 299.0574 [M-H-2H_2_O]^−^ (calcd. For C_16_H_13_O_7_^−^, 299.0570). The molecular formula along with NMR spectral data (Figure S25-31) suggested that **4** is 16 atomic mass units heavier than **1**, indicating the addition of a hydroxyl group. The physical and NMR spectral data closely resembled those of **1**. The ^1^H NMR spectrum for the A ring of **4** displayed protons and carbon signals including the junction (2,3-dihydroxypropanone) comparable to **1**. The observed change was from a 1,4-disubstitution in **1** to a 1,3,4-trisubstitution the B ring of **4**. The alternative oxygenation pattern was observed in the NMR data. The ^1^H-^1^H COSY, HMQC, and HMBC (Figure S28-S30), confirmed an aromatic B-ring trisubstituted system including characteristic signals: [*δ*_H_ 6.94 (1 H, d, *J* = 2.7 Hz, H-2)/ *δ*_C_ 114.6 (C-2); *δ*_H_ 6.81 (1 H, dd, *J* = 8.2, 2.7 Hz, H-6)/ *δ*_C_ 116.6 (C-6); *δ*_H_ 6.77 (1 H, d, *J* = 8.2 Hz, H-5)/*δ*_C_ 114.8 (C-5)], correspond to three aromatic protons of an ABX system. The magnitude of coupling constants showed that the methine at *δ*_H_ 6.81 (H-6) was *ortho* to the methine at *δ*_H_ 6.77 (H-5), and *meta* to the methine at *δ*_H_ 6.94 (H-2). Moreover ^13^, C NMR signals (Table 2) revealed three carbon signals at *δ*_C_ 128.4 (C-1), 145.0 (C-3), and 145.8 (C-4) were attributed to *sp*^2^ quaternary carbons. The proposed structure of the B ring was confirmed by HMBC (Fig. 2) i.e., from H-7 (*δ*_H_ 4.91)/ *δ*_C_ 128.4 (C-1), 114.6 (C-2), 119.6 (C-6); from H-6 (*δ*_H_ 6.81)/ *δ*_C_145.8 (C-4), 114.6 (C-2) and from H-5 (*δ*_H_ 6.77)/ *δ*_C_145.0 (C-3), 128.4 (C-1). The ^13^C NMR spectrum showed near overlap of C-2 (*δ*_C_ 114.6) and C-5 (*δ*_C_ 114.8), indicating that both carbon signals are almost equivalent. C-6 (*δ*_C_ 119.6) is shifted significantly downfield (Δ*δ*_C_ = + 4.8 ppm) in comparison with C-2 (*δ*_C_ 114.6) and C-5 (*δ*_C_ 114.8), because of the *para*-effect of the hydroxy group at C-3. Indeed, the ^1^H and ^13^C NMR spectra showed close similarity to those of **7**, isolated in this study and previously reported from *T. cilicicus*^17^. By comparison, a minor difference was observed between the two compounds in the A ring; C-4 was methoxylated in **4**. It appears that the comparable molecule was identified incorrectly and lacks chemical data supporting the hydroxyl group arrangement^4^. According to the above data, **4** was identified as (2S*,3S*)-1-(2,6-dihydroxy-4-methoxyphenyl)-3-(3,4-dihydroxyphenyl)-2,3-dihydroxypropan-1-one a new chalcanonoid given the trivial name pulincisone D.

**Table 2:** ^1^H NMR (600 MHz) and ^13^C NMR (125 MHz) spectral data of pulicisone D-F (**4-6**) in CD_3_OD (*δ* in ppm, *J* values in parentheses).

No	Pulincisone D (4)		Pulincisone E (5) ^b^		Pulincisone F (6)	
	δ_H_(J in Hz)	δ_C_	δ_H_(J in Hz)	δ_C_	δ_H_(J in Hz)	δ_C_
1		128.4		127.9		130.3
2	6.94 d (2.7)	114.6	7.04 d (2.7)	109.7	6.89 brs	113.4
3		145.0		146.8		145.2
4		145.8		146.8	6.75(overlap)	114.9*
5	6.77 d (8.2)	114.8	6.99 d (8.2)	114.7	6.78(overlap)	117.9*
6	6.81 dd (8.2, 2.7)	116.6	7.06 d (8.2, 2.7)	121.2		146.6
7	4.91 d (12.0)	83.9	5.01 d (12.0)	83.6	5.28 dd (12.4, 3.5)	79.3
8	4.51 d (12.0)	72.4	4.56 d (12.0)	72.5	2.70 dd (16.0, 3.5) 3.07 dd (16.0, 12.4)	42.4
9		197.6		196.0		196.9
1҆		101.3		100.9		102.7
2҆		163.0		163.0		163.3
3҆	6.00 d (2.7)	93.7	6.06 d (2.7)	93.8	6.01 d (2.7)	93.6
4҆		168.5		168.9		168.2
5҆	6.04 d (2.7)	94.7	6.11 d (2.7)	95.6	6.03 d (2.7)	94.4
6҆		163.7		163.7		163.9
4’-OMe	3.87 s	55.0	3.81 s	56.0	3.78 s	55.0
4-OMe			3.94 s	56.0		

Assignments were based on ^1^H-^1^H-COSY, HMQC and HMBC experiments.

^b^NMR data were run in CDCl_3_; *signals may be interchangeable.

Pulincisone E (**5**) was isolated as a colorless oil with an optical rotation of $\left[ a \right]_{D}^{{25}}$ = + 11.6 (*c* = 0.05, MeOH). The HRESIMS (Figure S40) exhibited a molecular ion peak at *m/z* 331.0818 [M-H-18]^−^ (calcd. for C_17_H_15_O_7_-, 331.0823), corresponding to a molecular formula of C_17_H_18_O_8_. Spectral data (Figure S33-38) were like **4**, except for the appearance of an additional methoxy group at *δ*_H_ 3.94 with a new signal at *δ*_C_ 56.0 (Table 2). NMR signal comparisons indicated that that the B-ring 3-OH in **4** was converted to a B-ring 3-OMe in **5**: [*δ*_H_ 6.94 (H-2)/ *δ*_C_ 114.6 (C-2); *δ*_H_ 6.81 (H-6)/ *δ*_C_ 119.6 (C-6),] for **4** and [*δ*_H_ 7.04 (H-2)/ *δ*_C_ 109.7 (C-2); *δ*_H_7.06 (H-6)/ *δ*_C_ 121.2 (C-6)] for **5**. A downfield shift of both protons H-2 and H-6 was observed with **5** by 0.10–0.25 ppm while C-5 shifted significantly upfield [Δ*δ*_C_ = 109.7 (**5**) – 114.6 (**4**) = -4.9 ppm] as anticipated by shielding of the mesomeric *ortho* effect. In contrast, C-6 shifted downfield [Δ*δ*_C_ = 121.2 (**5**) – 116.6 (**4**) = +4.6 ppm] due to *para* deshielding. Finally, the NOESY spectrum (Fig. 2 & S39) showed a correlation in the B ring between H-2 (*δ*_H_ 7.04) and the methoxy group (*δ*_H_ 3.94). All these assignments were compatible and led to the placement of the methoxy group at C-3. Another words, the replacement of a hydroxyl group in **4** by methoxy group in **5** at C-3. The^1^H NMR (Figure S33) showed a downfield singlet attributed to a phenolic proton at *δ*_H_ 11.2. The presence of a chelated hydroxy in an *ortho* position to the conjugated carbonyl was confirmed by the most downfield signal deshielded resonating at *δ*_C_ 196.0 (C-9) and *δ*_H_ 11.2 (s, 6’-OH). This observation was supported by HMBC experiments (Figure S37). Correlations were observed between the chelated hydroxyl group at *δ*_H_ 11.2 and the carbons *δ*_C_ 95.6 (C-5’), 100.9 (C-1’), and 163.7 (C-6’). This indicated that the chalconoid contained a phenolic hydroxyl *ortho* to the conjugated carbonyl located at C-6’.

An A ring aromatic AX spin system of ring suggested the presence of two *meta*-aromatic protons, one of them deshielded at *δ*_H_ 6.11 and the other shielded at *δ*_H_ 6.06. The de-shielded proton H-5 (*δ*_H_ 6.11) correlated with a phenolic resonance at *δ*_C_ 163.7, while the other shielded proton signal (*δ*_H_ 6.06) correlated with C-1’ (*δ*_C_ 100.9), C-2’ (*δ*_C_ 163.0), and C-4’ (*δ*_C_ 168.9) but not with C-6’ (Fig. 2). Thus, the chelating hydroxy group located at C-6’ was unequivocally assigned to the A ring with a *meta* coupling. The de-shielded signal of H-5’ (*δ*_H_ 6.11 and *δ*_C_ 95.6) indicated a periplanar relationship with the conjugated carbonyl at C-9. These data established **5** as (2S*,3S*)-1-(2,6-dihydroxy-4-methoxyphenyl)-2,3-dihydroxy-3-(4-hydroxy-3-methoxyphenyl) propan-1-one with a trivial name of Pulincisone E (Fig. 1).

Compound **6** was isolated as a light brownish oil with an optical rotation of $\left[ a \right]_{D}^{{25}}$ = − 9.9 (*c* = 0.05, MeOH). The HRESIMS (Figure S48) showed a molecular ion peak [M-H-18]^−^ at *m/z* 301.0708 (calcd. for C_16_H_13_O_6_^−^, 301.0712) corresponding to a molecular formula of C_16_H_16_O_7_ with nine degrees of unsaturation. A ketone group was observed by IR at 1705 cm^− 1^ and an NMR resonance at *δ*_C_ 196.9. The IR also exhibited an absorption band for a phenyl (2945 and 1499 cm^− 1^) and a hydroxyl (3398 cm^− 1^) group. A *β*-hydroxydihydrochalcone skeleton was revealed by the UV spectrum with absorption maxima at 225 and 288 nm^42^. Inspection of^1^H and ^13^C NMR data (Table 2 & S41-S43) showed signals suggestive of a hydroxydihydrochalcone derivative. The 1D and 2D NMR spectra (Figure S41-47) included: a carbinol, a methylene, and five olefinic protons. The only difference between this compound and the other isolated metabolites (**1**–**5**) was the appearance of a benzylic methylene at *δ*_C_ 42.7 and *δ*_H_ 2.70 (1 H, dd, *J* = 16.0, 3.5 Hz) with *δ*_H_ 3.07 (1 H, dd, *J* = 16.0, 12.4 Hz). Moreover, there is one unidentified coupling system with signals at *δ*_H_ 6.89 (1 H, brs, H-2), 6.75 (1 H, overlap, H-4), and 6.78 (1 H, overlap, H-5) in the B ring. The ^1^H-^1^ H COSY, HMQC, and HMBC (Figure S44-S46) confirmed an aryl B ring. The HBMC between [i] H-2/C-4, C-6, C-7, and [ii] H-4 with H-5/C-1, C-2, C-3, C-6 confirmed singlet signals at *δ*_H_ 6.89 (1 H, brs), 6.75 (1 H, overlap), and 6.78 (1 H, overlap) and were assigned to C-2, C-4, and C-5, respectively. The HMBC spectrum (Fig. 2 & S45) showed correlations between H_2_-8/C-7, C-1, C-9 and H-7/C-1, C-6 allowed the assignment of an aliphatic carbonyl to C-7, a benzylic methylene to C-8, and a ketone to C-9. Conformation of the relative configuration of the chiral center (C-7), which is a part of the ABX-type signals at [*δ*_H_ 2.70 (1 H, dd, *J* = 16.0, 3.5 Hz, H-8_a_); 3.07 (1 H, dd, *J* = 16.0, 12.4 Hz, H-8_b_); and 5.28 (1 H, dd, *J* = 12.4, 3.50 Hz, H-7); *δ*_C_ 42.4 (C-7), 79.3 (C-8)] was confirmed in comparison with (*αR*)-*α*,3,4,2’,4’-pentahydroxydihydrochalcone [*δ*_H_ : 2.73 (1 H, dd, *J* = 16.8, 3.0 Hz, H_a_-*β*), 3.00 (1 H, dd, *J* = 16.8, 13.2 Hz, H_b_-*β*), 5.30 (1 H, dd, *J* = 13.2, 3.0 Hz, H-*α*); *δ*_C_: 44.0 (C-*β*), 79.5 (C-*α*)]^43^, rhusopolyphenol F^25^ [*δ*_H_ : 2.66 (1 H, dd, *J* = 14.0, 1.0 Hz, H_a_-*β*), 3.33 (1 H, overlapped, H_b_-*β*), 5.79 (1 H, dd, *J* = 13.0, 1.0 Hz, H-*α*); *δ*_C_: 51.3 (C-*β*), 84.6 (C-*α*)], and with salsolol A^44^ [*δ*_H_ : 2.71 (1 H, dd, *J* = 17.1, 3.0 Hz, H_a_-*β*), 3.05 (1 H, *J* = 17.1, 12.6 Hz, H_b_-*β*), 5.31 (1 H, dd, *J* = 12.6, 3.0 Hz, H-*α*); *δ*_C_: 44.2 (C-*β*), 80.4 (C-*α*)]. The optical rotation of the isomers were recorded: rhusopolyphenol F^25^ ($\left[ a \right]_{D}^{{25}}$ = +19.4), salsolol A^44^ ($\left[ a \right]_{D}^{{25}}$ = -12.1), and **6** ($\left[ a \right]_{D}^{{25}}$ = − 9.9). Comparison of the above NMR data with an ABX-pattern as well as with optical rotations allowed for identifying a *β*-hydroxydihydrochalcone frame with a *R*-configuration of the asymmetric C-8. Thus, the relative configuration of **6** was (7*R*) based on comparison with the pro-*S* and pro-*R*. Based on this evidence, the structure was identified as (R*)-1-(2,6-dihydroxy-4-methoxyphenyl)-3-(3,6-dihydroxyphenyl)-3-hydroxypropan-1-one with a trivial name of pulincisone F (Fig. 1).

**Fig. 4:**
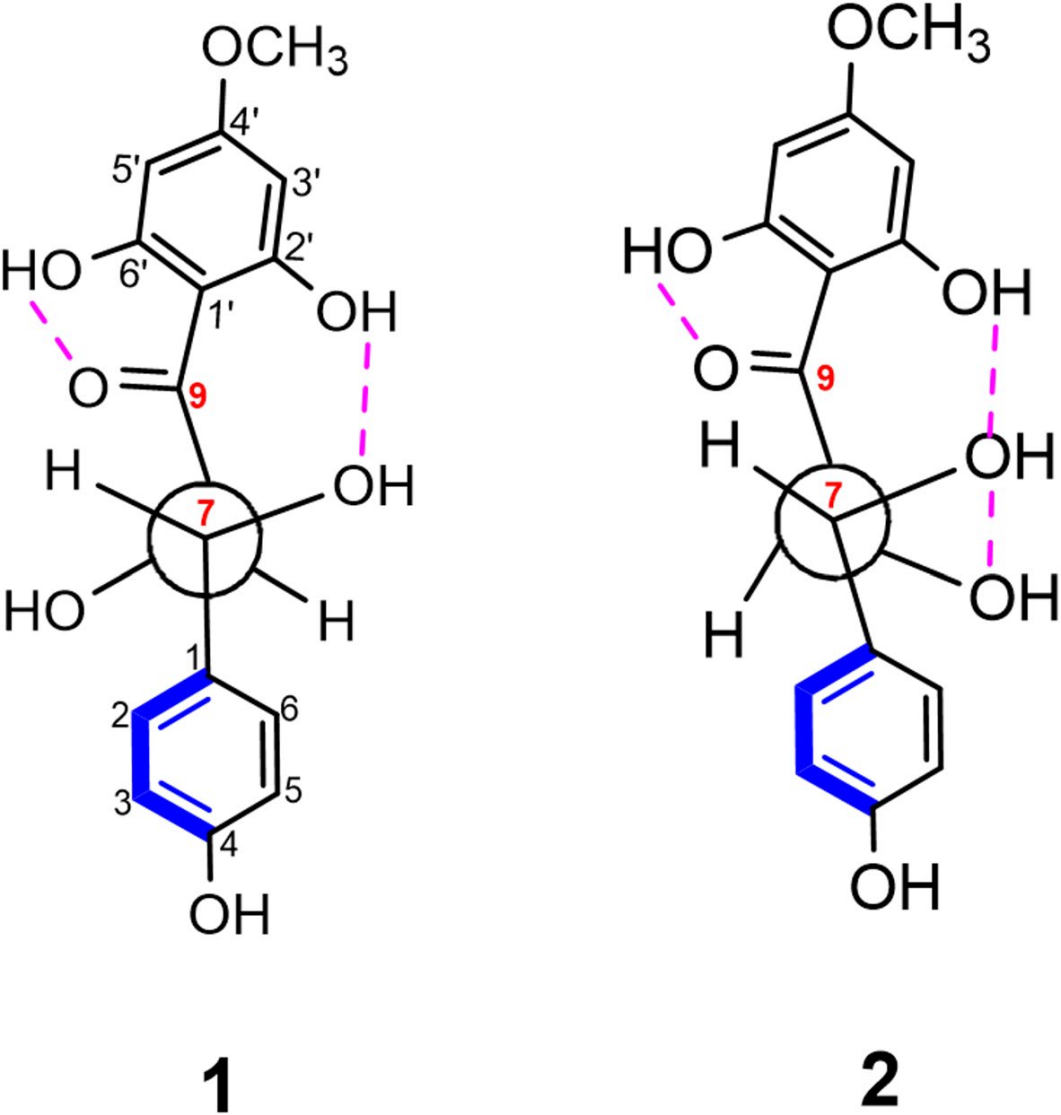
A Newman Projection of **1** and **2** onto C-7 and C-8 stereo centers. For **1**, the configuration is a *thero*-form, whereas for **2**, it is an *erythro*-form.

The relative configuration for 7,8-dihydroxychalcone (*β*’-chalcanone-*α*,*β*-diol) derivatives within a naliphatic three-carbon chain, has not been clear in a previous report^17^. Conflicting reports on the *β*’-chalcanone-*α*,*β*-diols pulichalconoid B and C, are presented^4^. Through space relationships for **1** from a Newman projection were consistent with HMQC and HMBC data, indicating connectivity for C-7 (*δ*_C_ 83.7) with H-7 (*δ*_H_ 4.95) and C-8 (*δ*_C_ 72.4) with H-8 (*δ*_H_ 4.53) (Fig. 4). An anisotropic effect for H-7 was proposed with A- and B-rings. Moreover, the electron withdrawing effect of the co-planar carbonyl group with H-7 (Fig. 4), causes de-shielding of H-7 and resulting in a down field shift in comparison with H-8. The spectral data indicates that H-7/H-8 doublets were incorrectly assigned in a previous analysis^4^. Compound **1** shows H-7 to have a *thero* configuration relationship with the vicinal coupling constant (*J* = 12.0 Hz), which can be predicted by the dihedral angle equal to 171.6º.

Many compounds can interaction with the solvent, especially if there are polar groups present^45^ e.g., the polarity of deuterated solvents, solvent-soluble interaction, and internal rotation. For this reason, the position of the phenolic hydroxy (6’-OH) in the aryl ring A of **4** was determined by comparison with literature data and by ^13^C NMR with ^1^H-^13^C HMBC spectroscopic analysis^44^. For example, the carbonyl carbon resonance at (C-9) exhibits dependence upon the absence or presence of a *peri*-substituent at (C-6’). In the case of 6’-hydroxylated, C-9 resonance absorbs at an appreciable de-shielded position of 195.6 -197.3 ppm, because of its involvement in chelation (intramolecular hydrogen bond interaction) with the hydroxy at the C-6’ position^24^. The loss of intramolecular hydrogen bond interaction with 6’-OMe is also responsible for the carbonyl absorption (C-9) to a 5–8 ppm upfield position^24^.

The arrangement of hydroxyl group at C-6’ in the A ring is also indicated by a correlation in the HMBC spectrum of **5**. The use of HMBC spectroscopy to solve such positional queries has been described previously^46^. Dreiding models also indicate co-planarity of the carbonyl group at C-9 and as the phenoxy group at C-6’ (Fig. 4). According to the above examination, the methoxy group in both compounds should be positionally revised to be attached to the C-3’ for the A ring, and the phenolic hydroxy should be in *ortho* position to the carbonyl group C-9. According to the above mentioned data both pulichalconoid B and C^4^ should be structurally revised to Pulicisone A (**1**) and D (**4**).

Except for **6** and **8** as *β*-hydroxychalcone and dihydrochalcone respectively, the other isolated compounds have two aryl rings joined by a three-carbon bridge, and an *α*,*β*-dihydroxy carbonyl system (**1**–**5**). Compound **7** was found in both diastereomeric forms (7*S*, 8*S*) and (7*S*, 8*R*). We were able to separate the diastereomers using HPLC, but it was not enough to prove absolute stereochemistry using X-rays or chemical transformation. These findings lead us to believe that *P. incisa* (Lam.) DC. produces approximately six times as many 7*S* and 8*S* diastereomers as the alternative one. The *trans*-isomer is typically thermodynamically more stable, making it the most common configuration for chalcones^47^.

### NQO1 inducer activities of isolated chalcones

To assess chalcone NQO1 inducing potency, Hepa1c1c7 cells were incubated for 48 h with increasing concentrations (0–50 µM) of the compound to be assayed; NQO1 activity was quantified using a Prochaska method. Compound **6** was the most potent inducer, with a CD value (concentration needed to double the NQO1 activity relative to the vehicle control) of 5.65 ± 0.6 µM (Table 3). Compounds **5**, **2**, and **3** had CD values of 6.75 ± 0.2, 7.10 ± 0.1, and 7.70 ± 0.2 µM, respectively. In contrast, **1**,** 4** and **8** showed significantly weaker inducing activities, with high CD values of 20.5 ± 0.5, 16.5 ± 0.5 and 20.5 ± 0.5 µM, respectively. No CD value was reached for **7** up to a concentration of 50 µM, suggesting that the presence of at least one methoxyl group in the A ring is important for cell permeability and/or inducing activity. Compound **5** contains a hydroquinone moiety, and since hydroquinones can undergo redox cycling to their quinone counterparts, which are known Nrf2/NQO1 inducers^48^this moiety could be also contributing to its inducing activity.

**Table 3:** NQO1 inducer potency for compounds **1**-**8**.

Compound	CD (µM)
1	20.5 ± 0.5
2	7.1±0.1
3	7.7±0.2
4	16.5 ± 0.5
5	6.75 ± 0.2
6	5.65±0.6
7	N.R
8	20.5±0.5
Sulforaphane (positive control)	0.25±0.1

Potency is expressed as the concentration of test sample needed to double NQO1-specific enzyme activity.

Early studies had shown that several Michael acceptor-bearing chalcones are NQO1 inducers^49^. Curiously, none of the isolated compounds described here bear Michael acceptor moieties, indicating that electrophilicity is not the primary mechanism by which these chalcones induce Nrf2 and strongly suggesting a cysteine-independent mechanism of action. Indeed, other studies have reported the chemo-preventive activity of dihydrochalcones via an activation of the Nrf2 pathway. Confusoside, a dihydrochalcone isolated from the medicinal plant *Anneslea fragrans*, was shown to activate the Nrf2/ARE pathway and increase the activity of related antioxidant proteins that resulted in the prevention of acetaminophen-induced liver injury in mice^50^. Moreover, the chemo-preventive potential of aspalathin, a dihydrochalcone isolated from *Aspalathus linearis* protected rat pancreatic β cells (INS1E β cell line) against the toxicity of streptozotosin, with chronic high glucose and H_2_O_2_^51^ levels by increasing the Nrf2/Keap1-dependent transcription of HO-1, NQO1 and SOD1 genes. In this study, aspalathin induced the protein expression of p62 (sequestosome-1), a protein that is both encoded by an Nrf2-target gene (SQSTM1) and is known to contribute to Nrf2 activation by competing with Nrf2 for binding to Keap1^52,53^.

### Induction of NQO1 protein expression

**Fig. 5:**
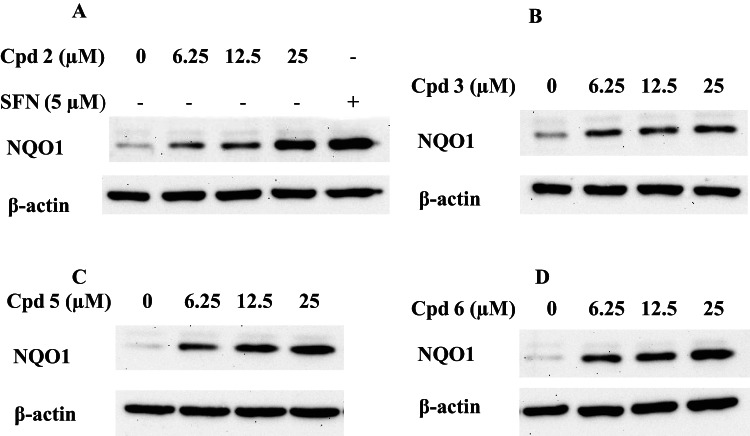
Western blots showing induction of NQO1 protein expression by indicated increasing concentrations of **2** (A), **3** (B), **5** (C) and **6** (D). Hepa1c1c7 cells were cultured and treated as described in the Experimental section. SFN: sulforaphane. Uncropped image of all blots shown is provided in supplementary information as Figure S49.

The active compounds with the lowest CD values (higher inducer activities, Table 3) were tested for their ability to induce NQO1 at the protein level by western blotting. Concentration-dependent upregulation of the NQO1 protein levels were observed, as shown in Fig. 5 (A-D & S49), in agreement with the enzyme activity data.

### *Insilico* molecular docking

**Fig. 6:**
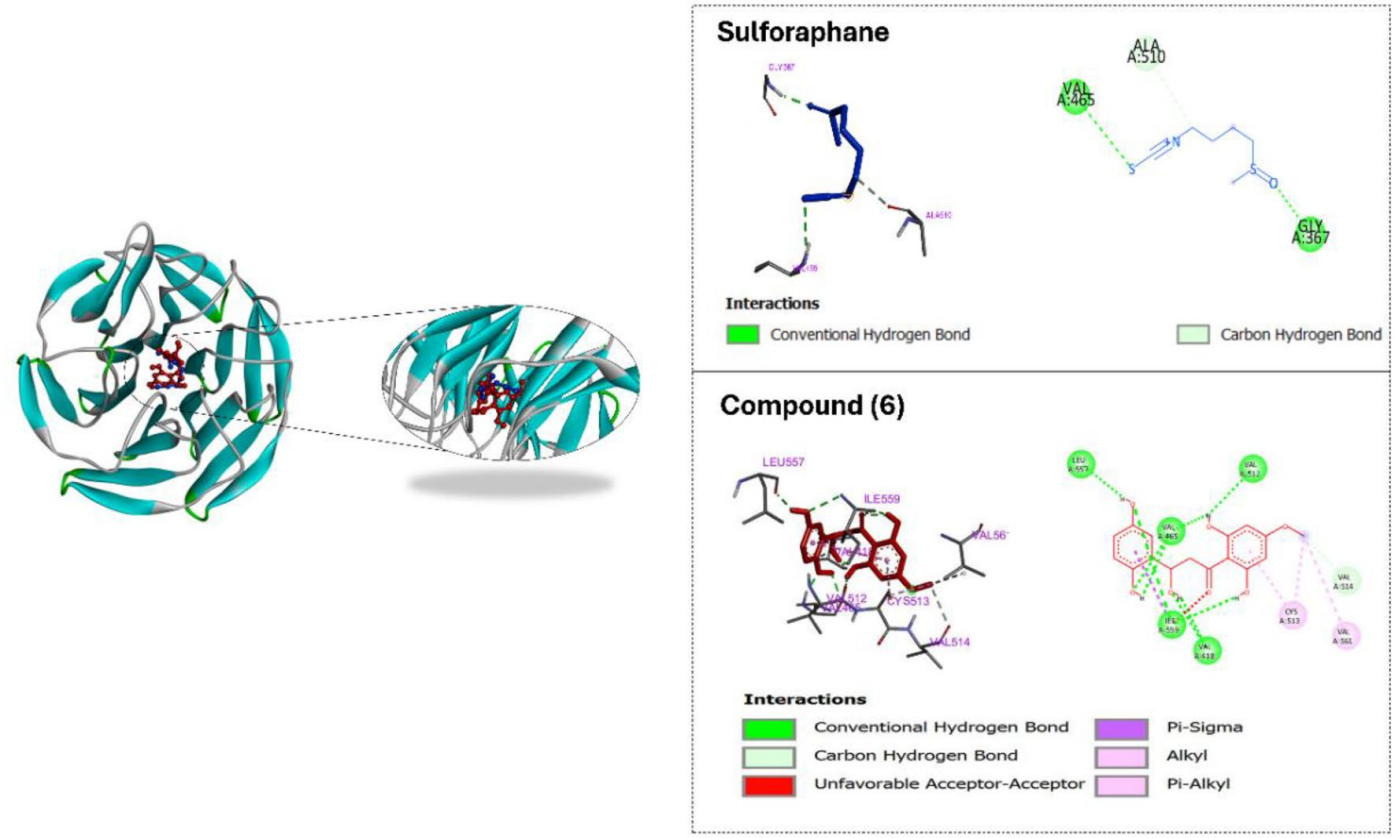
2D and 3D representations of the predicted binding modes inside the Kelch domain of the Keap1 crystal structure (PDB ID: 4IQK) for **6** (red), against NQO1 compared to the docked sulforaphane (blue) as a positive control.

Docking studies were performed with Keap1 to determine whether the metabolites would bind to Keap1 and activate Nrf2. It has been reported that compounds that interact with Keap1 alter conformation, causing Nrf2 accumulation and nuclear translocation, where Nrf2 binds to antioxidant response elements to reduce oxidative stress through the activation of a battery of antioxidant enzymes and proteins^54,55^. Compound **6** exhibited a superior docking score with Keap1 (− 8.26 kcal mol^−^) compared to sulforaphane (− 5.04 kcal mol^−^). Additionally, the inhibition constant (pK_i_) was 886.78 nM. The predicted binding interactions inside the Nrf2-binding site of Keap1 for **6** and sulforaphane are shown (Fig. 6). A channel-like shape in the middle of the structure of the Keap1-Kelch domain allowed **6** to fit into the structure to form a stable complex. Once the channel is occupied by **6**, the construction of the Keap1-Kelch domain is distorted and is predicted to no longer interact with NRF2; NRF2 exclusion results in NRF2 protein accumulation. According to the docking results **6** forms hydrophobic interactions with Cys513 and Val561 and hydrogen bonds with Val418, Val465, Val512, Val514, Leu557 and Ile559 (Fig. 6). The docking data are consistent with previous findings^56^ that although sulforaphane binds primarily to cysteine 151 in the *N*-terminal BTB-domain of Keap1, other cysteine modifications within Keap1 by sulforaphane, including cysteine 368 and cysteine 489 in the Kelch domain can be observed. While previous studies have shown that select chalcones that contain an electrophilic α, β-unsaturated carbonyl moiety, are NQO1 inducers^49,57^ this study testing newly isolated dihydrochalcone derivatives **2**,** 3**,** 5**, and **6** are NQO1 inducers, that may function by binding to the Kelch domain of Keap1 and prevent the interaction of Keap1 with Nrf2. Such interactions could explain how select chalcones can act as Keap1-Nrf2 protein-protein interaction inhibitors. This mechanism of action is distinct from that of the cysteine-modifying Michael acceptor-bearing chalcones.

## Experimental section

### Plant material

The aerial parts of *P. incisa* (Lam.) DC. were collected from Southern Sinai in Egypt during May 2017. A voucher specimen (SK-2017-15) has been deposited in the herbarium of Saint Katherine Protectorate, Egypt, with collection permission granted for scientific purposes by the Saint Katherine Protectorate.

### Extraction and isolation

The extraction and fractionation of the air-dried aerial parts of *P. incisa* (Lam.) DC. (1.0 kg) were previously described^12,58^. The extract was concentrated *in vacuo* at 45 °C to produce a dark brown residue (150 g). The material was purified on a silica gel column (6 × 120 cm) with a gradient of *n*-hexane up to 100% CH_2_Cl_2_ and CH_2_Cl_2_-MeOH up to 50% MeOH (3 L of each solvent combination). The *n*-hexane: CH_2_Cl_2_ fraction with ratio 1:3 (14.0 g) and CH_2_Cl_2_ (7.0 g) were added together according to their same chromatographic behavior using different solvent systems on TLC plates and then chromatographed on an ODS column (3 × 90 cm), eluted with 80% and 90% MeOH, and washed with 100% MeOH. To create two main fractions: fraction A (6.0 g) and fraction B (7.0 g), fraction A underwent further HPLC purification and MeOH: H_2_O elution (65–35% in 500 mL) by reverse-phase HPLC. The flow rate was set to 1.5 mL min^− 1^ and was at 0–70 min to afford **3** (20 mg, purity > 97% by HPLC) and **8** (14 mg, purity > 97% by HPLC). HPLC was used to purify fraction B, was eluted with MeOH: H_2_O (70:30%, 1000 mL) by reversed phase chromatography. The flow rate was set to 1.5 mL min^− 1^ and was at 0–70 min to afford **2** (10 mg, purity > 97% by HPLC), **1** (12 mg, purity > 97% by HPLC), and **6** (13 mg, purity > 97% by HPLC). The 5% MeOH fraction (8.5 g) was chromatographed on an ODS column (3 × 90 cm) eluted with 80% and 90% MeOH and washed with 100% MeOH. Fractions were obtained as one main fraction C (2.5 g), and HPLC was used to further purify C, which was eluted with MeOH: H_2_O (20: 80) by reversed phase chromatography. The flow rate was set to 2.0 mL min^− 1^ and was at 0–60 min. HPLC was further used to increase purity for **4** (9 mg, purity > 97% by HPLC), **5** (11 mg, purity > 97% by HPLC), and **7** (10 mg, purity > 97% by HPLC).

### General experimental procedures

Specific rotation was measured on a Horiba SEPA-300 digital polarimeter (5 cm) and IR data was collected from a Shimadzu FTIR-8100 spectrometer. The purified samples were conducted using a 6530B Accurate-mass-QTOF-MS mass spectrometer (Agilent Technologies, Inc., Santa Clara, CA, USA) equipped with an ESI-Jet Stream ion source through an HPLC/ESI-QTOF-MS system. Using tetramethyl silane as an internal standard, the ^1^H (600 MHz) and ^13^C (150 MHz) NMR spectra were run on a JEOL JNM-ECA 600 spectrometer. Compound purification for analytical and preparative separation was carried out using YMC-Pack ODS-A (250 × 4.6 mm i.d.) and (250 × 20 mm i.d.) columns, respectively. Purification was performed using a Shimadzu HPLC system furnished with a RID-10 A refractive index detector. Fuji Silysia Chemical, Ltd.‘s normal-phase silica BW-200 (Fuji Silysia Chemical, Ltd., 150–350 mesh) and Chromatorex’s ODS reverse phase DM1020T (Fuji Silysia Chemical, Ltd., 100–200 mesh) were used for chromatography separations (Fuji Silysia Chemical, Ltd., 100–200 mesh). Additionally, silica gel 60 F254 (Merck, 0.25 mm) and RP-18 WF254 were used (Merck, 0.25 mm) for TLC analysis with compound visualization by with H_2_SO_4_–MeOH (1: 9) spray followed by heating.

### Physicochemical constants

Pulincisone A (**1**): [(2S*,3S*)-1-(2,6-dihydroxy-4-methoxyphenyl)-2,3-dihydroxy-3-(4-hydroxyphenyl) propan-1-one] was isolated as a yellowish oil; $\:{\left[a\right]}_{D}^{25}$ = + 9.6 (*c* = 0.01, MeOH); UV (MeOH) λ_max_ nm (log ε): 213.3 (2.6), 287.2 (1.8) and 330.0 (0.4); IR (KBr) υ_max_ cm^− 1^: 3577, 3496, 3378, 3271, 2947, 1705, 1451, 1120, 1030; for ^1^H (600 MHz) and ^13^C (150 MHz) NMR spectroscopic data see Table 1 and Fig. S1-S7; HRESIMS *m/z* 301.0702 [M-H-18]^−^, (calcd. for C_16_H_13_O_6_^−^, 301.0712).

Pulincisone B (**2**): [(2R*,3S*)-1-(2,6-dihydroxy-4-methoxyphenyl)-2,3-dihydroxy-3-(4-hydroxyphenyl) propan-1-one] was isolated as a yellowish oil; $\:{\left[a\right]}_{D}^{25}$ = − 17.7 (*c* = 0.05, MeOH); UV (MeOH) λ _max_ nm (log ε): 213.3 (2.6), 287.2 (1.8) and 330.0 (0.4); IR υ_max_ cm^− 1^: 3450, 3378, 3271, 2947, 1705, 1451, 1120, 1030; for ^1^H (600 MHz) and ^13^C (150 MHz) NMR spectroscopic data see Table 1 & Figure S9-S15; HRESIMS *m/z* 301.0705 [M-H-18]^−^, (calcd. for C_16_H_13_O_6_^−^, 301.0712).

Pulincisone C (**3**): [(2S*,3S*)-1-(2,6-dihydroxy-4-methoxyphenyl)-2,3-dihydroxy-3-(4-methoxyphenyl) propan-1-one] was isolated as an amorphous yellowish powder; $\:{\left[a\right]}_{D}^{25}$ = + 19.1 (*c* = 0.02, MeOH); UV (MeOH) λ_max_ nm (log ε): 218 (3.9), 275 (2.5); IR (KBr) υ_max_ cm^− 1^: 3575, 3499, 3388, 3275, 2950, 1710, 1451, 1120, 1030; for ^1^H (600 MHz) and ^13^C (150 MHz) NMR spectroscopic data see Table 1& Figure S17-S23; HRESIMS *m/z* 301.0723 [M-H-MeOH]^−^, (calcd. for C_17_H_18_O_7_, 334.1053).

Pulincisone D (**4**): [(2S*,3S*)-1-(2,6-dihydroxy-4-methoxyphenyl)-3-(3,4-dihydroxyphenyl)-2,3-dihydroxypropan-1-one] was isolated as a brownish oil; $\:{\left[a\right]}_{D}^{25}$ = + 12.8 (*c* = 0.05, MeOH); UV (MeOH) λ_max_ nm (log ε): 214.4 (2.8), 278.9 (2.3) and 333.3 (0.5); IR (KBr) υ_max_ cm^− 1^: 3577, 3496, 3450, 3377, 3330, 3271, 2947, 1705, 1451, 1120, 1030, 690; for ^1^H (600 MHz) and ^13^C (150 MHz) NMR spectroscopic data see Table 2 & Figure S25-S31; HRESIMS *m/z* 299.0574 [M-H-2H_2_O]^−^ (calcd. for C_16_H_13_O_7_^−^, 299.0570).

Pulincisone E (**5**): [(2S*,3S*)-1-(2,6-dihydroxy-4-methoxyphenyl)-2,3-dihydroxy-3-(4-hydroxy-3-methoxyphenyl) propan-1-one] was isolated as a colorless oil; $\:{\left[a\right]}_{D}^{25}$ = + 11.6 (*c* = 0.01, MeOH); UV (MeOH) λ_max_ nm (log ε): 213.3 (2.9), 287.2 (2.2) and 330.0 (0.8); IR (KBr) υ_max_ cm^− 1^: 3579, 3496, 3455, 3381, 3333,3270, 2949, 1705, 1451, 1120, 1030, 690; for ^1^H (600 MHz) and ^13^C (150 MHz) NMR spectroscopic data see Table 2 & Figure S33-S39; HRESIMS *m/z* 331.0818 [M-H-18]^−^ (calcd. for C_17_H_15_O_7_^−^, 331.0823).

Pulincisone F (**6**): [(R*)-1-(2,6-dihydroxy-4-methoxyphenyl)-3-(3,4-dihydroxyphenyl)-2-hydroxypropan-1-one] was isolated as a light brownish oil; $\:{\left[a\right]}_{D}^{25}$ = − 9.9 (*c* = 0.05, MeOH); UV (MeOH) λ_max_ nm (log ε): 320 (3.31), 288 (3.44), 217 (4.11); IR (KBr) υ_max_ cm^− 1^:3398, 2945, 1705, 1499; for ^1^H (600 MHz) and ^13^C (150 MHz) NMR spectroscopic data see Table 2 & Figure S41-S47 ; HRESIMS *m/z* 301.0708 [M-H-18]^−^ (calcd. for C_16_H_13_O_6_^−^, 301.0712).

### Biological assay

#### Cell culture

Murine hepatoma Hepa1c1c7 cells (obtained from ATCC^®^, USA) were grown in α-MEM supplemented medium with 10% (v/v) heat- and charcoal-inactivated fetal bovine serum. Cells were routinely maintained in a humidified incubator at 37 °C, 5% CO_2_.

#### NQO1 inducer activity (Prochaska assay)

A quantitative NQO1 microtiter plate test as employed, following a previously published method^59,60^. In 96-well plates, 10,000 cells were seeded per well and left overnight. On the next day, the cell culture medium was substituted with fresh medium that contained chalcones (**1**–**8**). The cells were then subjected to a further 48-h incubation. Eight replicate wells were used to evaluate five serial dilutions (ranging from 0 to 50 µM) for each compound. The compounds were initially made as concentrated solutions in dimethyl sulfoxide (DMSO) and subsequently diluted in the cell culture medium at a ratio of 1:1,000. After a 48-h exposure, the cells were lysed for 30 min at a temperature of 25 °C using digitonin (0.8 g per liter, pH 7.8). Cellular lysates were utilized to assess the enzymatic activity of NQO1, employing menadione as the substrate. The protein concentrations in each well were measured using the BCA protein assay from Thermo Scientific, UK. Sulforaphane, a potent classical NQO1 inducer^61^ was used as a positive control.

### Western blotting for NQO1

For analysis of NQO1protein levels, Hepa1c1c7 cells (overnight incubated monolayers of 3 × 10^5^ cells/well grown in 6-well plates) were treated with three concentrations of chalcones (6.25, 12.5 and 25 µM) or vehicle (0.1% DMSO). Sulforaphane (positive control) was also used as an inducer of NQO1. After 24 h, monolayers were washed with ice-cold DPBS and lysed^62^. Cell suspensions were then sonicated on ice (30% amplitude-for 5 sec). Sonicates were centrifuged at 4 °C (10,000×g for 10 min). Supernatants (cytosolic proteins) were collected for electrophoresis. Lysate proteins were loaded onto 10% polyacrylamide gel and separated by electrophoresis using a Biorad Tetra Cell (Biorad, USA) for 90 min at 110 volts. Resolved proteins were transferred to nitrocellulose membrane by blotting a Biorad Trans blot module for 60 min at 100 volts. Membranes were blocked using 5% non-fat milk in Tris buffered saline tween 20 (TBST) for 1 h. at room temp with gentle shaking, and probing with primary NQO1 (Abcam, UK) or *β*-actin (Thermofisher Scientific, USA) antibodies at 4 °C/overnight with gentle rolling on a tube roller. The next day, membranes were washed 3 × 5 min with TBST. Membranes were then probed with the corresponding HRP-conjugated secondary antibodies for 1 h. The membranes were washed as above with TBST. Enzyme chemi-luminescence (Pierce, Thermofisher Scientific, USA) was used to visualize protein bands. The bands were captured using a UVP Biospectrum imager (UVP in the UK, which was driven by the Visionworks Acquisition and Analysis software package (Analytik Jena, USA) (version 8.20.17096.9551).

### Molecular docking

The structure of the flavonoid compounds, as well as positive control, sulforaphane for NQO1 induction, were downloaded from PubChem database (https://pubchem.ncbi.nlm.nih.gov/*)* as SDF files. Avogadro (https://avogadro.cc/*)* software was used to convert SDF files to PDB format. The PDB file for Keap1 crystal structure (PDB ID: 4IQK) was downloaded from the Protein Data Bank (http://www.rcsb.org/pdb/*).* Docking was performed three times independently to calculate mean values and standard deviations of lowest binding energies and predicted inhibition constants (pKi). The representation and graphical analyses were performed using the BIOVIA Discovery Studio Visualizer software. In this study Autodock Tools (1.5.6rc316) were used to prepare protein structures and molecular docking simulations. Docking parameters were set to 250 runs and 2,500,000 energy evaluations for each cycle. Docking was performed by Autodock4, as previously described using a Lamarckian Algorithm^63^.

## Conclusion

Six highly oxygenated *β*’-chalcanone-*α*,*β*-diolnovelchalconoids, pulincisone A-F (**1**–**6**), along with two recognized analogues 3,4,2’,4’,6’,7,8-heptahydroxy-7(8)-dihydro-chalcone **(7)** and 4,2’,6’-trihydroxy-4’-methoxydihydrochalcone **(8)**, were isolated from the methylene chloride/methanol (1:1) extract of air-dried aerial parts of wild *P. incisa* (Lam.) DC., and identified using physical and spectroscopic data measurements. NMR data analysis and comparison of physical and spectral data with those of previously reported literature compounds, complete ^1^H and ^13^C NMR assignments were made. The chalcone hydroxyl derivative, pulincisone F **(6)**, is the most potent NQO1 inducer among the isolated chalcone derivatives and exhibited superior docking score with Keap1 compared to sulforaphane, indicating that it may inhibit the Keap1-Nrf2 protein-protein complex and act as a chemo-preventive agent. These findings underscore the potential of *P. incisa* (Lam.) DC. as a source of novel chemo-preventive agents and provide a basis for further studies (Scheme 1).

## Supplementary Information

Below is the link to the electronic supplementary material.

Supplementary Material 1

## Data Availability

All data generated or analysed during this study are included in this published article [and its supplementary information files].
