# Supplementary material for: Chalcone constituents pulincisones A-F isolated from Pulicaria incisa with NQO1 inducer activity
Source: Sci Rep. 2025 Aug 20;15:30632. doi: 10.1038/s41598-025-15999-2 (PMC12368085; doi:10.1038/s41598-025-15999-2)
Supplement: Supplementary file 1 — Supplementary Material 1 [file 41598_2025_15999_MOESM1_ESM.docx]

**Supporting Information**

**Chalcone Constituents Pulincisones A-F isolated from *Pulicaria incisa* with NQO1 Inducer activity**

Ahmed R. Hamed^a^*, Tarik A. Mohamed^aɸ^, Sally A. Abdel-Halim^a^, Mohamed H. Abd El-Razek^bɸ^, Sayed A. El-toumy^c^, Maureen Higgins^d^, Krystyna Skalicka-Woźniak^e^, Albena T. Dinkova-Kostova^d^, Paul W. Pare^f^, Mahmoud M. Sakr^g^, Mohamed-Elamir F. Hegazy^a^*

^a^ Chemistry of Medicinal Plants Department, National Research Centre, 33 El-Bohouth St., Dokki, Giza, 12622, Egypt.

^b^ Natural Compounds Chemistry Department, National Research Centre, 33 El-Bohouth St., Dokki, Giza, 12622, Egypt.

^c^ Department of Chemistry of Tannins, National Research Centre, 33 El-Bohouth St., Dokki, Giza, 12622, Egypt.

^d^Jacqui Wood Cancer Centre, Division of Cellular and Systems Medicine, School of Medicine, University of Dundee, Dundee, UK, Dundee DD1 9SY, Scotland, UK.

^e^ Department of Chemistry of Natural Products, Medical University of Lublin, Lublin, Poland.

^f^ Department of Chemistry & Biochemistry Texas Tech University, Lubbock, TX 79409 USA.

^g^ Department of Plant Biotechnology, Institute of Genetic Engineering and Biotechnology, National Research Centre, 33 El-Bohouth St., Dokki, Giza 12622, Egypt.

**^*^Corresponding Authors: Ahmed R. Hamed**, Email: ar.hamed@nrc.sci.eg Tel.: +20 1090221769; Tel.: +20-1127539989; **Mohamed-Elamir F. Hegazy**, Email: me.fathy@nrc.sci.eg (M.-E.F.H.); Tel.: +20-33-371-635.

^ɸ^ Authors with equal contribution.

**KEYWORDS:** Pulicaria incisa, Asteraceae, dihydrochalcone, chemo-preventive, antioxidant, NQO1.

**ABSTRACT:** **Purpose:** Previously unknown chalcones such as *β*'-chalcanone-*α*,*β*-diols and a *β*-hydroxydihydrochalcone, named pulincisone A-F as well as known chalcone analogs 3,4,2',4',6',7,8-heptahydroxy-7(8)-dihydrochalcone (**7**) and 4,2',6'-trihydroxy-4'-methoxydihydrochalcone (**8**) were isolated from *Pulicaria incisa* (Lam.) DC. and assayed for cancer preventative activity. **Methods**: Structures were identified by spectroscopic methods, including HRESIMS, 1D and 2D NMR experiments. Pulincisone A and B, are epimers, at stereocenter C-8. A structural revision resulted in pulicisone A (**1**) and D (**4**) replacing pulichalconoids B and C, that were previously reported from the same species. Isolated chalcones were assessed for chemopreventive potential as inducers of NAD(P)H:quinone oxidoreductase 1 (NQO1). **Results:** Compounds **2**, **3**, **5**, and **6** induced NQO1, using a Prochaska assay. NQO1 protein expression was detected by Western blotting analysis. Compound **6** among four active compounds had the highest potency as NQO1 inducers. Molecular docking analysis of the NQO1 Keap1 Kelch domain suggests Nrf2-dependent induction. **Conclusion:** These data indicate that pulincisone F (**6**) is a promising bioactive antioxidant (NQO1-inducer) with superior docking scores with the Keap1-Nrf2 complex compared to the reference NQO1 inducer sulforaphane (−8.26 versus −5.08 kcal mol^-^, respectively).

**CONTENTS**

| Supporting data | Page |
| --- | --- |
| Figure S1: ^1^H NMR spectrum (CD_3_OD, 600 MHz) of Pulincisone A (**1**) | 5 |
| Figure S2: ^13^C NMR spectrum (CD_3_OD, 150 MHz) of Pulincisone A (**1**) | 6 |
| Figure S3: DEPT spectrum of Pulincisone A (**1**) | 7 |
| Figure S4: ^1^H-^1^H COSY spctrum of Pulincisone A (**1**) | 8 |
| Figure S5: HMBC spectrum of Pulincisone A (**1**) | 9 |
| Figure S6: HMQC spectrum of Pulincisone A (**1**) | 10 |
| Figure S7: NOESY spectrum of Pulincisone A (**1**) | 11 |
| Figure S8: HR-ESI MS spectrum of Pulincisone A (**1**) | 12 |
| Figure S9: ^1^H NMR spectrum (CD_3_OD, 600 MHz) of Pulincisone B (**2**) | 13 |
| Figure S10: ^13^C NMR spectrum (CD_3_OD, 150 MHz) of Pulincisone B (**2**) | 14 |
| Figure S11: DEPT spectrum of Pulincisone B (**2**) | 15 |
| Figure S12: ^1^H-^1^H COSY spctrum of Pulincisone B (**2**) | 16 |
| Figure S13: HMBC spectrum of Pulincisone B (**2**) | 17 |
| Figure S14: HMQC spectrum of Pulincisone B (**2**) | 18 |
| Figure S15: NOESY spectrum of Pulincisone B (**2**) | 19 |
| Figure S16: HR-ESI MS spectrum of Pulincisone B (**2**) | 20 |
| Figure S17: ^1^H NMR spectrum (CD_3_OD, 600 MHz) of Pulincisone C (**3**) | 21 |
| Figure S18: ^13^C NMR spectrum (CD_3_OD, 150 MHz) of Pulincisone C (**3**) | 22 |
| Figure S19: DEPT spectrum of Pulincisone C (**3**) | 23 |
| Figure S20: ^1^H-^1^H COSY spctrum of Pulincisone C (**3**) | 24 |
| Figure S21: HMBC spectrum of Pulincisone C (**3**) | 25 |
| Figure S22: HMQC spectrum of Pulincisone C (**3**) | 26 |
| Figure S23: NOESY spectrum of Pulincisone C (**3**) | 27 |
| Figure S24: HR-ESI MS spectrum of Pulincisone C (**3**) | 28 |
| Figure S25: ^1^H NMR spectrum (CD_3_OD, 600 MHz) of Pulincisone D (**4**) | 29 |
| Figure S26: ^13^C NMR spectrum (CD_3_OD, 150 MHz) of Pulincisone D (**4**) | 30 |
| Figure S27: DEPT spectrum of Pulincisone D (**4**) | 31 |
| Figure S28: ^1^H-^1^H COSY spctrum of Pulincisone D (**4**) | 32 |
| Figure S29: HMBC spectrum of Pulincisone D (**4**) | 33 |
| Figure S30: HMQC spectrum of Pulincisone D (**4**) | 34 |
| Figure S31: NOESY spectrum of Pulincisone D (**4**) | 35 |
| Figure S32: HR-ESI MS spectrum of Pulincisone D (**4**) | 36 |
| Figure S33: ^1^H NMR spectrum (CDCl3, 600 MHz) of Pulincisone E (**5**) | 37 |
| Figure S34: ^13^C NMR spectrum (CDCl3, 150 MHz) of Pulincisone E (**5**) | 38 |
| Figure S35: DEPT spectrum of Pulincisone E (**5**) | 39 |
| Figure S36: ^1^H-^1^H COSY spctrum of Pulincisone E (**5**) | 40 |
| Figure S37: HMBC spectrum of Pulincisone E (**5**) | 41 |
| Figure S38: HMQC spectrum of Pulincisone E (**5**) | 42 |
| Figure S39: NOESY spectrum of Pulincisone E (**5**) | 43 |
| Figure S40: HR-ESI MS spectrum of Pulincisone E (**5**) | 44 |
| Figure S41: ^1^H NMR spectrum (CD_3_OD, 600 MHz) of Pulincisone F (**6**) | 45 |
| Figure S42: ^13^C NMR spectrum (CD_3_OD, 150 MHz) of Pulincisone F (**6**) | 46 |
| Figure S43: DEPT spectrum of Pulincisone F (**6**) | 47 |
| Figure S44: ^1^H-^1^H COSY spctrum of Pulincisone F (**6**) | 48 |
| Figure S45: HMBC spectrum of Pulincisone F (**6**) | 49 |
| Figure S46: HMQC spectrum of Pulincisone F (**6**) | 50 |
| Figure S47: NOESY spectrum of Pulincisone F (**6**) | 51 |
| Figure S48: HR-ESI MS spectrum of Pulincisone F (**6**) | 52 |
| Figure S49 : Uncropped blots of Figure 5 (A-D) | 53 |

Figure S1: ^1^H NMR spectrum (CD_3_OD, 600 MHz) of Pulincisone A (**1**)

Figure S2: ^13^C NMR spectrum (CD_3_OD, 150 MHz) of Pulincisone A (**1**)

Figure S3: DEPT spectrum of Pulincisone A (**1**)

Figure S4: ^1^H-^1^H COSY spctrum of Pulincisone A (**1**)

Figure S5: HMQC spectrum of Pulincisone A (**1**)

Figure S6: HMBC spectrum of Pulincisone A (**1**)

Figure S7: NOESY spectrum of Pulincisone A (**1**)

Figure S8: HR-ESI MS spectrum of Pulincisone A (**1**)

Figure S9: ^1^H NMR spectrum (CD_3_OD, 600 MHz) of Pulincisone B (**2**)

Figure S10: ^13^C NMR spectrum (CD_3_OD, 150 MHz) of Pulincisone B (**2**)

Figure S11: DEPT spectrum of Pulincisone B (**2**)

Figure S12: ^1^H-^1^H COSY spctrum of Pulincisone B (**2**)

Figure S13: HMBC spectrum of Pulincisone B (**2**)

Figure S14: HMQC spectrum of Pulincisone B (**2**)

Figure S15: NOESY spectrum of Pulincisone B (**2**)

Figure S16: HR-ESI MS spectrum of Pulincisone B (**2**)

Figure S17: ^1^H NMR spectrum (CD_3_OD, 600 MHz) of Pulincisone C (**3**)

Figure S18: ^13^C NMR spectrum (CD_3_OD, 150 MHz) of Pulincisone C (**3**)

Figure S19: DEPT spectrum of Pulincisone C (**3**)

Figure S20: ^1^H-^1^H COSY spctrum of Pulincisone C (**3**)

Figure S21: HMBC spectrum of Pulincisone C (**3**)

Figure S22: HMQC spectrum of Pulincisone C (**3**)

Figure S23: NOESY spectrum of Pulincisone C (**3**)

Figure S24: HR-ESI MS spectrum of Pulincisone C (**3**)

Figure S25: ^1^H NMR spectrum (CD_3_OD, 600 MHz) of Pulincisone D (**4**)

Figure S26: ^13^C NMR spectrum (CD_3_OD, 150 MHz) of Pulincisone D (**4**)

Figure S27: DEPT spectrum of Pulincisone D (**4**)

Figure S28: ^1^H-^1^H COSY spctrum of Pulincisone D (**4**)

Figure S29: HMBC spectrum of Pulincisone D (**4**)

Figure S30: HMQC spectrum of Pulincisone D (**4**)

Figure S31: NOESY spectrum of Pulincisone D (**4**)

Figure S32: HR-ESI MS spectrum of Pulincisone D (**4**)

Figure S33: ^1^H NMR spectrum (CDCl3, 600 MHz) of Pulincisone E (**5**)

Figure S34: ^13^C NMR spectrum (CDCl3, 150 MHz) of Pulincisone E (**5**)

Figure S35: DEPT spectrum of Pulincisone E (**5**)

Figure S36: ^1^H-^1^H COSY spctrum of Pulincisone E (**5**)

Figure S37: HMBC spectrum of Pulincisone E (**5**)

Figure S38: HMQC spectrum of Pulincisone E (**5**)

Figure S39: NOESY spectrum of Pulincisone E (**5**)

Figure S40: HR-ESI MS spectrum of Pulincisone E (**5**)

Figure S41: ^1^H NMR spectrum (CD_3_OD, 600 MHz) of Pulincisone F (**6**)

Figure S42: ^13^C NMR spectrum (CD_3_OD, 150 MHz) of Pulincisone F (**6**)

Figure S43: DEPT spectrum of Pulincisone F (**6**)

Figure S44: ^1^H-^1^H COSY spctrum of Pulincisone F (**6**)

Figure S45: HMBC spectrum of Pulincisone F (**6**)

Figure S46: HMQC spectrum of Pulincisone F (**6**)

Figure S47: NOESY spectrum of Pulincisone F (**6**)

Figure S48: HR-ESI MS spectrum of Pulincisone F (**6**)

Figure S49: Uncropped Chemiluminescence image of blots shown on Figure 5 (A-D). Image background was adjusted to show the blot membrane strips and developed bands at the same time. Cropped blots shown in Figure 5 (A-D) are inverted versions of the bands shown inside each red square. We added text label inside each red square to further indicate each compound included in Figure 5 (A-D). Red arrows refer to the target protein bands.
